# Characterization and phylogenetic analysis of the complete mitochondrial genome of *Stephnometra indica* (Pelmatozoa: Crinoidea)

**DOI:** 10.1080/23802359.2019.1627923

**Published:** 2019-07-11

**Authors:** Shaobo Ma, Huixian Zhang, Xin Wang, Jianping Yin, Pingping Shen, Qiang Lin

**Affiliations:** a CAS Key Laboratory of Tropical Marine Bio-resources and Ecology, South China Sea Institute of Oceanology, Chinese Academy of Sciences, Guangzhou, P. R. China;; b Guangdong Provincial Key Laboratory of Applied Marine Biology, South China Sea Institute of Oceanology, Chinese Academy of Sciences, Guangzhou, P. R. China;; c University of Chinese Academy of Sciences, Beijing, P. R. China

**Keywords:** *Stephnometra indica*, mitochondrial genome, phylogenetic analysis

## Abstract

The crinoid *Stephnometra indica* inhabits coral reefs and surrounding waters in the western Pacific Ocean. In this study, the complete mitochondrial genome of *S. indica* (15,878 bp) was confirmed to contain 13 protein-coding genes, 22 tRNA genes, and 2 rRNA genes. The gene order of *S. indica* was identical to that of previously published Crinoidea. The nucleotide composition of the *S. indica* mitogenome was biased toward A + T nucleotide 73.68%. There were four unassigned regions (UASs) found in *S. indica* and all four UAS regions were AT-biased. The phylogenetic relationship illustrated that *S. indica* is more closely related to *Antedon mediterranea* within the family Crinoidea.

Crinoids are generally considered the earliest diverged group of echinoderms, and have inhabited the oceans for more than 500 million years (Smith 1988; Wada and Satoh 1994; Littlewood and Smith 1995; Ausich et al. 1999). Furthermore, crinoids are considered potentially important model organisms for evolutionary developmental biology, because they retained all three primitive coelom-related compartments among extant echinoderms (Marchand 1994; Mooi and David 1997, 1998). In recent years, Stephnometra *indica*’s habitats have suffered degradation from global warming, ocean acidification (OA). However, the researches of *S. indica* are still poorly investigated. So this study will be helpful to effective management and conservation strategies for this species.

The sample of *S. indica* was collected from Meiji Island in the Spratly Islands, South China Sea (9°55′6354″ N, 115°33′4168″ S) and stored at Sample center of South China Sea Institute of Oceanology, CAS (SCSIO-NS-MJ-00354). A small amount of tissue was cut from the body and frozen in liquid nitrogen until DNA extraction. Ten pairs of primer were designed from conservative regions based on the alignment of complete mitochondrial genomes available within the family Crinoidea. We sequenced the complete mitochondrial genome of *S. indica* with the Gene Bank accession number MF966246. The mitogenome of *S. indica* was a typical circular DNA molecule, with 15,878 bp in length, contained 22 tRNA genes, 13 protein-coding genes (PCGs), 2 ribosomal genes, and 4 unassigned sequences (UASs). Twenty of 37 genes were encoded by the H-strand, and another 17 genes were encoded by the L-strand. All 22 tRNA secondary structures had typical cloverleaf structure except tRNA^Ser (AGY)^ missing the whole dihydrouridine arm. The gene order of *S. indica* is identical to that of previously published crinoids, including the position of the *nad4L* gene, which was considered a distinguishing feature between echinoderms and vertebrates (Jacobs et al. 1988; Scouras and Smith 2006). The overall base compositions of A, T, C, and G are 25.86, 47.83, 15.40, and 10.96%, respectively, with an obvious AT bias feature (73.70%). There were four UASs in *S. indica* with an obvious A + T-rich features, three of the UASs were previously recognized in other crinoids (UAS I, UAS II, and UAS III) (Scouras and Smith 2006), the additional UAS IV was identified in *S. indica*, and was also found in the mitogenome of *Gymnocrinus richeri* (Scouras and Smith 2006). Some reports speculated UAS IV region appears by a duplication event (Perseke et al. 2008).

Phylogenetic analyses of *S. indica* and other echinoderms were conducted based on 13 mitochondrial PCGs using the maximum-likelihood (ML) method, and *Balanoglossus clavigerus* (Hemichordata) was selected as an outgroup. ML analyses show that all five echinoderm classes were recovered as monophyletic groups in each case and species of Crinoidea formed an independent and well-supported clade, and then clustered with the families Holothuroidea and Ophiuroidea. Stephnometra *indica* is more closely related to *Antedon mediterranea* within the family Crinoidea. This result provides important data for the phylogenetic relationship between crinoids and echinoderms. Molecular data partitioning of different crinoid species gives essentially congruent results. To figure out complex phylogenetic relationships between echinoderms, further sequence data is required.

## Figures and Tables

**Figure 1.**
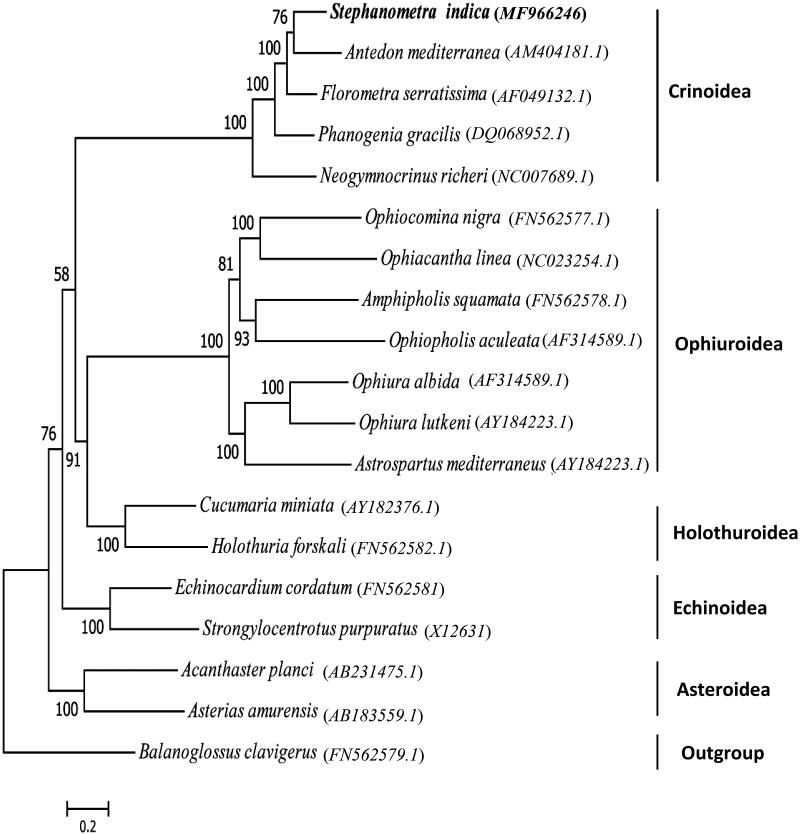
Phylogenetic analyses of *S. indica* and other echinoderms were conducted based on 13 mitochondrial PCGs using a maximum likelihood (ML) method, *Balanoglossus clavigerus* (Hemichordata) was used as an outgroup (Perseke et al. 2008).
